# Suckling or non‐suckling? Sensory characterization of commercialized lamb meat according to feeding

**DOI:** 10.1002/fsn3.3604

**Published:** 2023-08-25

**Authors:** Iñaki Etaio, Leire Bravo‐Lamas, Francisco José Pérez‐Elortondo, Luis Javier R. Barron, Noelia Aldai

**Affiliations:** ^1^ Laboratorio de Análisis Sensorial Euskal Herriko Unibertsitatea (LASEHU) Vitoria‐Gasteiz Spain; ^2^ Lactiker Research Group, Department of Pharmacy & Food Sciences University of the Basque Country (UPV/EHU), Centro de Investigación Lascaray Ikergunea Vitoria‐Gasteiz Spain

**Keywords:** descriptive sensory references, market survey, ovine meat, sensory characterization, suckling lamb

## Abstract

Consumption of meat from suckling lambs is typical in some regions. However, sensory differences between meat from suckling and non‐suckling lambs are barely described in previous studies. The objectives of the present study were (a) to develop a method to describe the sensory characteristics of lamb meat, including the development of sensory references for odor, flavor, and texture attributes; and (b) to study the sensory differences between lamb meat commercialized as “suckling lamb” and that commercialized without this designation. Twenty‐three sensory attributes were selected, and their corresponding sensory references were developed. A detailed procedure to evaluate the samples was also set up. This methodology was used to characterize samples (*n* = 48) from a survey of lamb meat from different types of stores (*n* = 23). Half of the samples were commercialized as sucking lamb and the other half (older lambs) without this indication. Samples were bought in two seasons (May and December) to consider possible seasonal effects. Samples were evaluated in triplicate by 12 trained assessors. Data were analyzed by ANOVA (*p* ≤ .05). Apart from how long juiciness was maintained (“maintained juiciness”), all the selected attributes were appropriate to discriminate between samples. Lamb meat sold as “suckling” did not differ from the other lamb samples in odor and flavor, but there were clear differences in texture attributes: meat sold as suckling lamb was tenderer and juicier, with higher crumbliness, and with lower fibrousness, chewiness, and residue than non‐suckling lamb meat. Several sensory characteristics, mainly related to odor and flavor, varied according to the season.

## Graphical abstract

- Representative lamb meat samples commercialized as “suckling lamb” and without this indication were collected in 23 stores in two different seasons (May and December).
- A method (including sensory references) was developed for the sensory description of the samples, and a panel was trained to evaluate them.
- Several sensory differences were found according to the feeding and to the season.

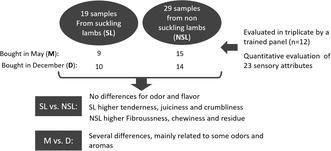

## 1 INTRODUCTION

Ovine meat consumption worldwide is low compared to other meats such as beef, pork, or poultry. In 2021, world sheep consumption averaged 1.78 kg per capita (as retail weight) (OECD/FAO, 2021) with marked regional differences: 1.53 kg in Europe, 0.57 kg in North America, 0.60 kg in Latin America, 2.14 kg in Africa, 4.99 kg in Oceania, and 1.95 kg in Asia. These differences were also considerable between countries, ranging from 0.03 in Thailand to 7.95 kg in Kazakhstan. Essentially, consumption is relatively regular in some countries, while in others it is mainly associated with celebrations and festivities (Garnier, 2010). In the Basque Country and Navarre Communities in northern Spain in particular, the consumption of ovine meat is mainly related to celebrations, especially the Christmas season (Ellies‐Oury et al., 2022). Lamb consumption in 2021 made up 2.21% and 3.76% of the total fresh meat consumed in the Basque Country and Navarre, respectively, with a consumption of 1.12 and 1.93 kg per capita (MAPA, 2022). Moreover, traditionally consumed ovine meat comes from very young animals, usually suckling lambs (Villalva et al., 2013).

Sheep production in the aforementioned regions is primarily dedicated to cheese‐making (“Idiazabal” and “Roncal” Protected Designations of Origin) which is mainly based on the extensive or semi‐extensive production of autochthonous Latxa breed. In this sense, suckling lamb meat is considered a high‐quality by‐product which generates extra income for local shepherds. However, this local meat competes with both national (mainly Castile, Aragón, Extremadura) and international (France, UK, Ireland, New Zealand) imported lamb/sheep meats (Mediano et al., 2009).

Sensory characteristics of lamb meat described by trained panels have been reported in several studies (Carlucci et al., 1999; Gasperi et al., 2005; Karamichou et al., 2007; Komprda et al., 2012; Sañudo, Nute, et al., 1998). The degree of coincidence between attributes used to describe lamb samples in these studies can be considered to be relatively high. Nevertheless, the number of studies describing the use of sensory references to homogenize the concepts associated with the different terms and to train assessors is very scarce (Gasperi et al., 2005; Ruiz de Huidobro et al., 2001). Moreover, although there is a study describing the sensory characteristics of meat from Latxa and Rasa lamb breeds from the Aragon region (Gorraiz et al., 2000), there is no comprehensive characterization of lamb meat commercialized and consumed in the Basque Country and Navarre.

Suckling lambs are characterized by being fed only with mother's (ewe) milk. They are slaughtered at 3–5 weeks, providing carcass weights of 5.5–7.5 kg (Beriain et al., 2000; Panea et al., 2013; Sañudo, Sanchez, & Alfonso, 1998), while lamb meat commercialized without the “suckling” designation usually comes from older animals that weigh more when slaughtered. These animals are normally fed on other feeds rather than ewe´s milk. At selling points, however, it is often difficult to guarantee that all lamb meat sold with the “suckling lamb” designation comes from suckling lambs fed exclusively with ewe's milk.

Meat from suckling lambs is usually described as softer in texture and smoother in flavor compared to meat from older animals that had forage or have had concentrate included in their diets after the initial suckling stage. However, studies dealing with the possible sensory differences between these two types of lamb meat are scarce (Carlucci et al., 1999; Gorraiz et al., 2000; Panea et al., 2013). Certain studies have described the sensory characteristics of suckling lamb meat but did not compare it to other lamb meat (Revilla et al., 2021; Ruiz de Huidobro et al., 1998, 2001).

Therefore, the main objectives of the present study were (1) to develop a method to evaluate lamb meat, including sensory references, and (2) to characterize lamb meat commercialized in the Basque Country and Navarre Communities on the basis of a survey performed in large stores and smaller butcher shops in different cities. Furthermore, the possible differences between lamb meat sold as “suckling lamb” and meat sold without this denomination have been considered to complement the sensory description. Samples were surveyed in two different seasons in order to consider the possible variation in the meat sensory characteristics throughout the year.

## 2 MATERIALS AND METHODS

### 2.1 Lamb meat samples

Table 1 shows the origin and characteristics of lamb chops used in the study. Lamb chop samples were purchased from 23 preselected stores in Vitoria‐Gasteiz, Donostia‐San Sebastian, Bilbo‐Bilbao, and Iruñea‐Pamplona in two different periods (May—season 1, and December—season 2). Ten stores were medium to large supermarkets (in one of them two different samples were obtained), and 13 stores were small butcher shops. Among the latter, one of the butcher shops in each city was located in a traditional fresh produce market with many different little food shops, as this was considered an important grocery acquisition point. The ratio between large stores and butcher shops (11/13) was established considering published data regarding the places where ovine and goat meat is bought in Spain (Langreo, 2008): 49.6% of the sold meat value in butchers' shops and 39.8% in medium to large supermarkets, while direct purchase from producers and self‐supply were relatively minor. According to the sales presentation in each store, lamb chops were purchased packaged in trays or cut directly from the carcass. Information about origin and feeding of the animal was collected from labels on trays, from signs in the stores and/or from the seller. As indicated previously, the objective of the present study is to characterize lamb meat sold in the Basque Country and Navarre Communities on the basis of a survey, while also considering the season and the detail as to whether it was being sold as suckling lamb or not.

**TABLE 1:** Commercial lamb meat samples analyzed in the study.

Sample code^a^	Season	Store type	City	Feeding^b^	Lamb geographical origin^c^	Meat presentation^d^
1‐1NS	May	Supermarket	Vitoria‐Gasteiz	Non‐suckling	Spain	TP
1‐2NS	December	Supermarket	Vitoria‐Gasteiz	Non‐suckling	Spain	TP
2‐1S	May	Supermarket	Vitoria‐Gasteiz	Suckling	Burgos	DCFC
2‐2S	December	Supermarket	Vitoria‐Gasteiz	Suckling	Navarre	DCFC
3‐1NS	May	Supermarket	Vitoria‐Gasteiz	Non‐suckling	Aragon	TP
3‐2NS	December	Supermarket	Vitoria‐Gasteiz	Non‐suckling	Aragon	TP
4‐1NS	May	Supermarket	Vitoria‐Gasteiz	Non‐suckling	Spain	TP
4‐2NS	December	Supermarket	Vitoria‐Gasteiz	Non‐suckling	Spain	DCFC
5‐1S	May	Supermarket	Vitoria‐Gasteiz	Suckling	Spain	TP
5‐2S	December	Supermarket	Vitoria‐Gasteiz	Suckling	“Slaughtered in Spain”	TP
6‐1NS	May	Supermarket	Vitoria‐Gasteiz	Non‐suckling	Navarre	DCFC
6‐2NS	December	Supermarket	Vitoria‐Gasteiz	Non‐suckling	Navarre	DCFC
7‐1NS	May	Supermarket	Vitoria‐Gasteiz	Non‐suckling	–	TP
7‐2NS	December	Supermarket	Vitoria‐Gasteiz	Non‐suckling	–	TP
8‐1NS	May	Supermarket	Oiartzun/Donostia‐San Sebastián	Non‐suckling	Aragon	TP
8‐2NS	December	Supermarket	Oiartzun/Donostia‐San Sebastián	Non‐suckling	Aragon	DCFC
9‐1S	May	Supermarket	Donostia‐San Sebastián	Suckling	Castile and Leon	DCFC
9‐2S	December	Supermarket	Donostia‐San Sebastián	Suckling	Castile and Leon	DCFC
10‐1NS	May	Supermarket	Bilbo‐Bilbao	Non‐suckling	Zamora	DCFC
10‐2S	December	Supermarket	Bilbo‐Bilbao	Suckling	Zamora	TP
11‐1S	May	Butcher's shop	Vitoria‐Gasteiz	Suckling	Navarre	DCFC
11‐2NS	December	Butcher's shop	Vitoria‐Gasteiz	Non‐suckling	Basque Country Community	DCFC
12‐1NS	May	Supermarket	Antsoain/Iruñea‐Pamplona	Non‐suckling	–	TP
12‐2NS	December	Supermarket	Antsoain/Iruñea‐Pamplona	Non‐suckling	–	TP
13‐1S	May	Butcher's shop	Vitoria‐Gasteiz	Suckling	Burgos	DCFC
13‐2S	December	Butcher's shop	Vitoria‐Gasteiz	Suckling	Palencia	DCFC
14‐1S	May	Butcher's shop	Donostia‐San Sebastián	Suckling	Castile and Leon	DCFC
14‐2S	December	Butcher's shop	Donostia‐San Sebastián	Suckling	Castile and Leon	DCFC
15‐1NS	May	Butcher's shop	Bilbo‐Bilbao	Non‐suckling	Castile and Leon	DCFC
15‐2NS	December	Butcher's shop	Bilbo‐Bilbao	Non‐suckling	Castile and Leon	DCFC
16‐1S	May	Butcher's shop	Iruñea‐Pamplona	Suckling	Navarre	DCFC
16‐2S	December	Butcher's shop	Iruñea‐Pamplona	Suckling	Navarre	DCFC
17‐1NS	May	Butcher's shop	Vitoria‐Gasteiz	Non‐suckling	Burgos	DCFC
17‐2NS	December	Butcher's shop	Vitoria‐Gasteiz	Non‐suckling	“Born in Extremadura and grown in Basque Country”	DCFC
18‐1NS	May	Butcher's shop	Vitoria‐Gasteiz	Non‐suckling	Araba	DCFC
18‐2S	December	Butcher's shop	Vitoria‐Gasteiz	Suckling	Araba	DCFC
19‐1NS	May	Butcher's shop	Donostia‐San Sebastián	Non‐suckling	Navarre	DCFC
19‐2NS	December	Butcher's shop	Donostia‐San Sebastián	Non‐suckling	Navarre	DCFC
20‐1NS	May	Butcher's shop	Donostia‐San Sebastián	Non‐suckling	Basque Country Community	DCFC
20‐2NS	December	Butcher's shop	Donostia‐San Sebastián	Non‐suckling	Basque Country Community	DCFC
21‐1S	May	Butcher's shop	Bilbo‐Bilbao	Suckling	Castile and Leon	DCFC
21‐2S	December	Butcher's shop	Bilbo‐Bilbao	Suckling	Castile and Leon	DCFC
22‐1S	May	Butcher's shop	Bilbo‐Bilbao	Suckling	Burgos	DCFC
22‐2S	December	Butcher's shop	Bilbo‐Bilbao	Suckling	Burgos	DCFC
23‐1NS	May	Butcher's shop	Iruñea‐Pamplona	Non‐suckling	Navarre	DCFC
23‐2NS	December	Butcher's shop	Iruñea‐Pamplona	Non‐suckling	Navarre	DCFC
24‐1NS	May	Butcher's shop	Iruñea‐Pamplona	Non‐suckling	Navarre	DCFC
24‐2NS	December	Butcher's shop	Iruñea‐Pamplona	Non‐suckling	Navarre	DCFC

^a^ First number of the code: specific store (1–24, in the case of 1 and 3, they are the same store); 2nd number: season (1: May/2: December); S/NS: suckling lamb/non‐suckling lamb.

^b^ According to the label (in tray‐packaged chops) or to the signs in the store stand (in chops directly cut from the carcass).

^c^ As indicated in the label or by the seller (in chops directly cut from the carcass).

^d^ TP, tray‐packaged/DCFC, directly cut from the carcass.

Lamb chops for the survey were transported to the laboratory in refrigerated conditions. Chops from each sample were distributed in three groups (three replicates), wrapped in plastic film for food use and kept frozen at −28°C until analysis.

### 2.2 Assessors

Assessors were recruited from interested people who had previously passed selection tests and basic training tests in sensory analyses of food. These tests checked possible sensory inabilities, in addition to candidates' sensibility and ability to describe their perceptions. Tests were carried out according to a normalized technical procedure described in Pérez Elortondo et al. (2007).

The panel to develop the method for lamb meat evaluation and to evaluate the samples was initially attended by 15 assessors, most of them with experience in sensory analysis of specific products. One member left the panel during the method development phase, and two other members could not attend the sessions during season 2. As a result, only data from the 12 assessors attending seasons 1 and 2 (8 women and 4 men, average age of 38) were considered.

### 2.3 Attribute selection, reference development, panel training, and qualification

**FIGURE 1:**
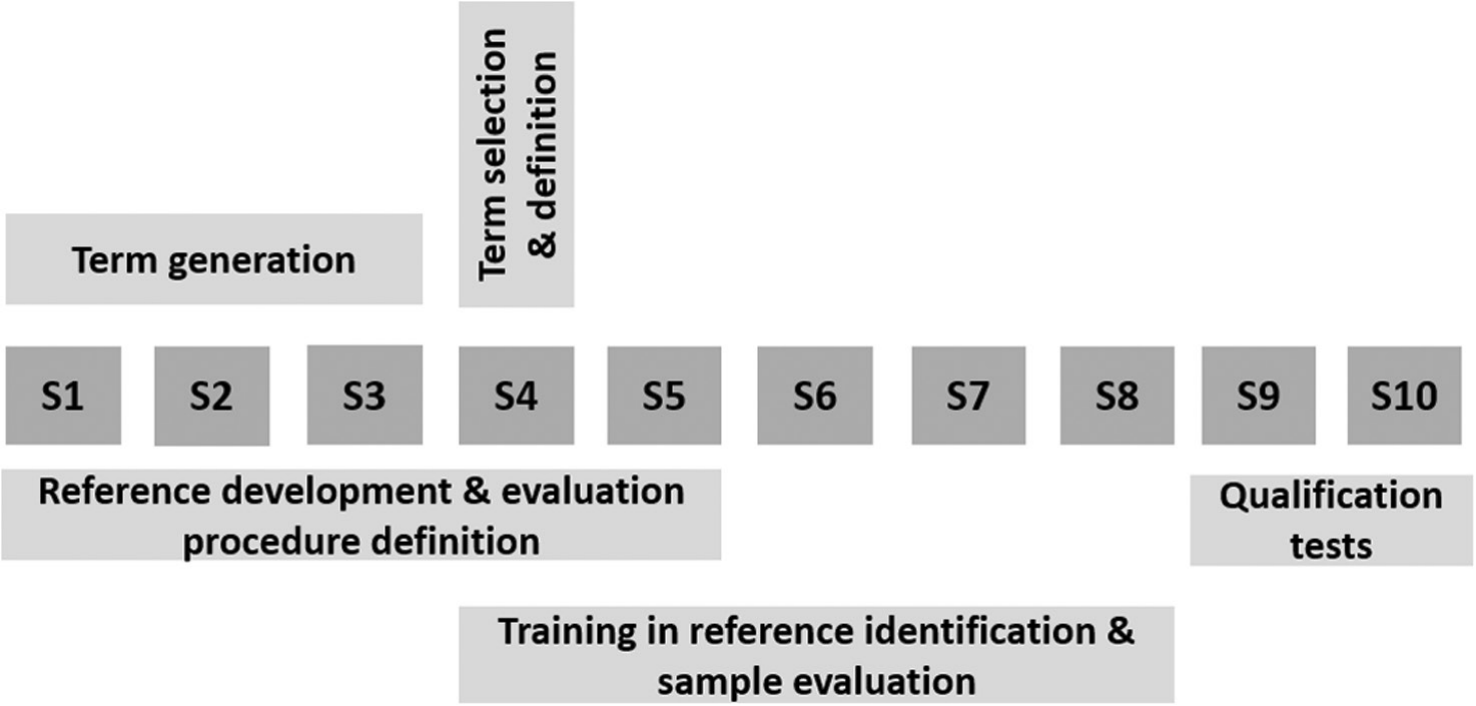
Procedure used to develop the sensory methodology and to get ready the panel during the 10 sessions (S).

The procedure to develop an evaluation method and to get the panel ready was obtained during eight sessions of approximately 90 min (Figure 1). Terms to describe the odor, texture, and flavor of lamb meat samples were generated in the first three sessions by comparing, sample by sample, four pairs of different samples in order to indicate the similarities and differences, and by direct description of seven samples. Samples used for term generation and panel training were commercial samples from different stores.

**FIGURE 2:**
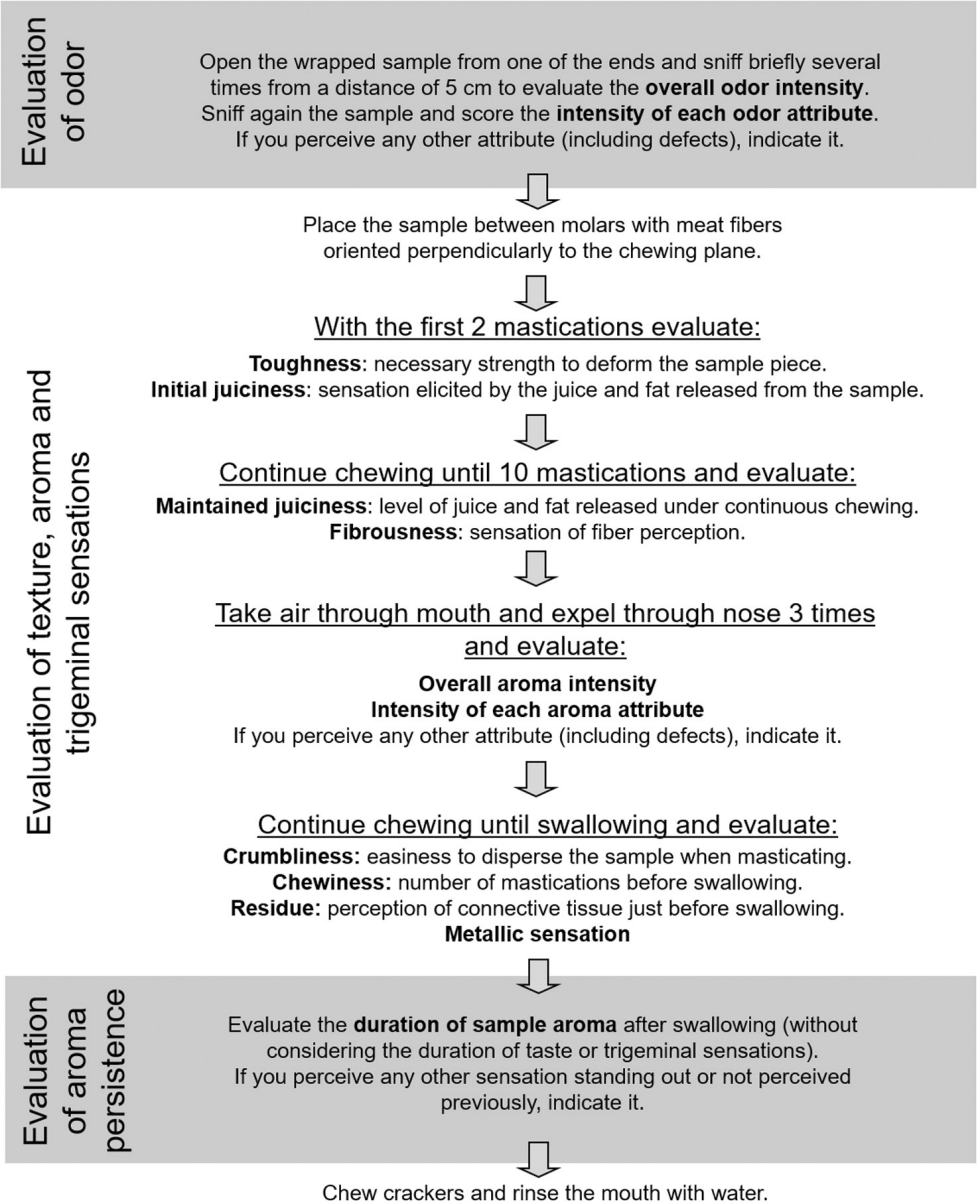
Methodology for sensory evaluation of samples.

The terms generated were discussed within the panel, and typical attributes mentioned in other lamb meat studies were taken into consideration. The attributes to assess, with their corresponding definitions, were established after session 3. In order to facilitate discussion and clarify concepts, possible references were presented to the assessors so that they could reproduce the selected terms more easily. Sensory references were developed throughout the first five sessions, by presenting, discussing, and modifying them until reaching an agreement about their appropriateness. The list of odor, texture, and flavor attributes and the composition of the corresponding references are summarized in Table 2. A continuous 10 cm scale anchored at points 0 and 10 and with an additional cm at each end was used to score the intensity of each attribute. Except for odor/aroma intensity, references to odor/aroma and trigeminal sensations were qualitative. To ease the process of grading the intensity of attributes perceived in the samples, the following indications were included in the scale: “Not perceived at all” (cm 0), “hardly perceived (doubtful presence)” (cm 2), “slightly perceived” (cm 4), “quite clearly perceived but not high intensity” (cm 6), “clearly perceived and high intensity” (cm 8), and “very high intensity” (cm 10). In the case of cut grass, urine/cowshed, and rancid odor/aroma, although references were developed and assessors were trained to identify them, it was decided not to include specific scales in the scorecard since these attributes are very infrequent (urine/cowshed and rancid odor/aroma are defects). An option of “Other attributes/defects” was included in the scorecard to indicate the perception of these or other strange sensations. A detailed methodology for sample evaluation was also defined throughout the first sessions (Figure 2).

**TABLE 2:** Composition and preparation procedure of sensory references for lamb meat evaluation.

Attribute	Reference composition	Conservation
Odor/aroma intensity	MB diluted at 50% in water (odor/aroma intensity reference located in cm 8 on the scale)	Frozen
Cooked meat odor/aroma	The same as for odor/aroma intensity	Frozen
Fat odor/aroma	1.5 g of adipose tissue from lamb chop heated in microwave oven for 2 min at defrost power (200 W) in 20 mL of MB diluted at 20% in water	Frozen
Liver odor/aroma	80 g of calf liver boiled in 400 mL of water for 15 min. Homogenized with a mixer and lyophilized.	Lyophilized sample refrigerated.
	0.4 g of the lyophilized sample in 20 mL of water	Reference frozen.
Blood odor/aroma	3 mL of blood in 20 mL of water	Blood and reference frozen
Dairy odor/aroma	5 mL of cow whole milk +1 g of butter in 20 mL of MB diluted at 20% in water	Frozen
Forage odor/aroma	0.2 g of grinded dehydrated alfalfa in 20 mL of MB diluted at 20% in water	Frozen
Cut grass odor/aroma	0.2 g of fresh cut grass in 20 mL of MB diluted at 20% in water	Prepared in each session
Urine/cowshed odor/aroma	0.05 g of lyophilized sheep urine reconstituted in 20 mL of MB diluted at 20% in water	Frozen
Rancid odor/aroma	1.5 g of adipose tissue from lamb chop heated at 50°C for 2 weeks	Refrigerated
Toughness	Pork lean (Apis brand) in 1.5 × 1.5 × 1 cm pieces: tender (cm 2 on the scale)	Prepared in each session
	Fuet (Casa Tarradellas brand) in 1.5 × 1.5 × 1 cm pieces: tough (cm 8 on the scale)	
Initial juiciness	Frankfurt sausage (Campofrío brand) in 1.5 × 1.5 × 1 cm pieces: little juicy (cm 1 on the scale)	Prepared in each session
	Golden apple in 1.5 × 1.5 × 1 cm pieces: very juicy (cm 9 on the scale)	
Maintained juiciness	For maintained juiciness several indications were pointed out on the scale: *much lower than initial juiciness* (cm 0), *quite lower than initial juiciness* (cm 3.33), *bit lower than initial juiciness* (cm 6.66), and *similar to initial juiciness* (cm 10).	
Fibrousness	Frankfurt sausage (Campofrío brand) in 1.5 × 1.5 × 1 cm pieces: not fibrous at all (cm 0 on the scale) Canned tuna, raw (Calvo brand) in pieces: very fibrous (cm 8 on the scale)	Prepared in each session
Crumbliness	Canned tuna, raw (Calvo brand) in pieces: little crumbly (cm 2 on the scale)	Prepared in each session
	Frankfurt sausage (Campofrío brand) in 1.5 × 1.5 × 1 cm pieces: very crumbly (cm 8 on the scale)	
Chewiness	For chewiness, the number of mastications before swallowing the samples was marked on the scale: 15 (cm 0), 20 (cm 2), 25 (cm 4), 30 (cm 6), 35 (cm 8), and 40 (cm 10).	
Residue	Fuet (Casa Tarradellas brand) in 1.5 × 1.5 × 1 cm pieces: quite high residue (cm 7 on the scale)	Prepared in each session
Metallic sensation	1 g of ferrous sulfate heptahydrated (Riedel‐deHaën) in 400 mL of MB diluted at 30% in water	Prepared in each session
Aroma persistence	For global aroma persistence each cm on the 10 cm scale corresponds to one second (scale ranging from 0 to 10 s).	

Abbreviations: MB, Commercial meat broth (Gallina Blanca brand).

In sessions 4 to 8, assessors were trained in reference identification and in lamb meat description by evaluating 18 additional samples. After training and before starting the evaluation of the study samples, qualification tests were carried out to verify the suitable performance of the panel. Participants were assessed on their ability to repeatedly identify the sensory references (within sessions) and their reproducibility (identifying them repeatedly between sessions), in addition to their discrimination ability when scoring the sensory attributes.

### 2.4 Sample distribution, preparation, and serving

**FIGURE 3:**
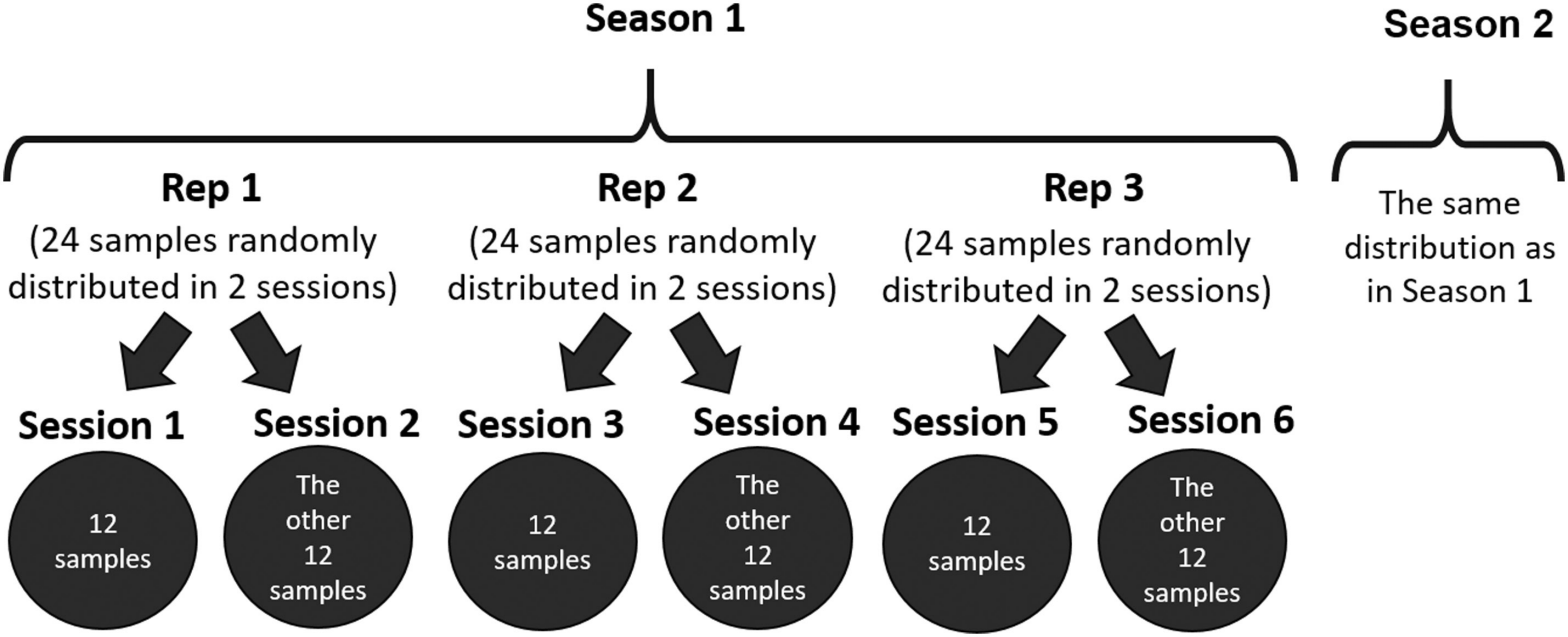
Distribution of samples throughout the evaluation sessions.

The 24 samples corresponding to each season were evaluated in triplicate throughout six sessions, with samples of each replication distributed randomly between two sessions (12 samples per session), as indicated in Figure 3.

The day before evaluation, chops were thawed in a refrigerator (4°C) and placed at room temperature 3 h before cooking. From each chop, the *Longissimus thoracis et lumborum* muscle was extracted, surrounding fascia was removed, and, when necessary, the muscle was cut into pieces of approximately 1.5 cm length × 1.5 cm width × 1 cm thickness. Each piece was wrapped in aluminum foil and codified (codes had been randomly established previously). The evaluation order of samples was also randomly established by a Latin Square distribution and was different for each assessor. The 12 samples to be evaluated in each session were distributed in two sets of six samples, in order to avoid the excessive drying of the meat and in order to maintain a break between the two sets. Sample distribution among assessors was randomized across the two sets to minimize a possible cooking effect.

The six samples of the corresponding set of each assessor were wrapped together in an aluminum foil package, with the assessor's name written on each. The packages of the first set were cooked in a preheated (180°C) conventional oven (Franke Planet Pyro HPP‐1046 Inox, Franke, Switzerland) at an internal temperature of 80°C, as measured with a portable thermometer (TM‐946, Lutron Electronic Enterprise Co., Ltd., Taiwan) equipped with K‐type thermocouples. Six thermocouples were inserted in the geometric center of additional meat pieces (with the same dimensions as the samples) and placed in the packages together with the samples. When meat pieces reached 80°C, packages were taken out of the oven and placed in a heater (heat diffusion through glass microspheres; Indoterm, Indo, Sant Cugat, Barcelona). Heaters were placed in each booth to maintain the temperature of the samples until evaluation.

### 2.5 Sample evaluation

Previous to sample evaluation, sensory references of odor, texture, and metallic sensations were tested by the assessors in a discussion room. First, to serve as a panel calibration, a lamb meat sample prepared as previously described was evaluated in booths, and the scores given by the assessors were discussed in the discussion room. Thereafter, evaluation of the 12 samples was carried out in booths maintained at 21 ± 2°C and under red/green illumination to avoid color bias. FIZZ software (Biosystèmes, Couternon, version 2.40 H) was used for data acquisition. Each assessor evaluated the samples following the order shown on the computer screen (randomly established for each assessor). A waiting time of 30 s before the code of the next sample appeared, was established. After evaluating the first six samples, assessors left the laboratory for 5–10 min, while samples that had already been evaluated were removed and the package with the next six samples was placed in the heater devices.

Samples were evaluated, and scores for each attribute were assigned according to the procedure described in Figure 2, which was also detailed in the evaluation guide provided to each assessor. Assessors were instructed to chew salt‐free crackers and rinse the mouth with water between samples to remove residual sensations.

### 2.6 Data analysis

To study the attributes that distinguish the various samples, a general linear model of analysis of variance (ANOVA) was carried out over the scores given to the 48 samples by the 12 assessors attending seasons 1 and 2. The model used was the following:

$$\begin{array}{c} Y=\mu +\text{Season}+\text{Assessor}+\text{Sample}\left(\text{Season}\right)+\text{Session}\left(\text{Season}\right)\\ \;+{\text{Assessor}}^{*}\text{Sample}\left(\text{Season}\right) \end{array}$$

Season was included as a fixed factor and assessor; sample (season) and session (season) were included as random effects. Sample and session factors were placed under the season factor since samples evaluated in season 1 were not the same as the ones evaluated in season 2. Similarly, sessions 1 to 6 corresponded to season 1, while sessions 7 to 12 corresponded to season 2.

To study the effect of feeding (suckling lambs vs. non‐suckling) and season (samples purchased in May vs. December), a four‐way ANOVA was carried out with the average scores of the panel for each repetition (three scores for each sample) according to the following model:

$$\begin{array}{c} Y=\mu +\text{Feeding}+\text{Season}+\text{Store}\left(\text{Feeding}\right)+\text{Session}\left(\text{Season}\right)\\ \;+{\text{Feeding}}^{*}\text{Season} \end{array}$$

Feeding and season were fixed factors, and store (feeding) and session (season) were included as random effects.

Store factor was included under the feeding factor since samples bought in each store usually belong to the same feeding type in both seasons. In this sense, data from stores selling lamb's meat where the feeding type changes from one season to the other (samples 10‐1NS/10‐2S, 11‐1S/11‐2NS, and 18‐1NS/18‐2S; see Table 1) were removed from the analysis. Session factor was included in the analysis within the season factor since sessions 1 to 6 corresponded to season 1 and sessions 7 to 12 to season 2.

ANOVA analysis was run with IBM SPSS Statistics 24.0 (IBM Corporation, NY, USA), and *p* ≤ .05 was considered indicative of statistically significant differences.

Principal component analysis (PCA) was performed with the mean calculated from the three replication means of the panel for each of the 48 samples using Pearson correlation coefficients. XLStat 2016.1 (Addinsoft, Paris, France) was used for PCA. Attribute loading and mean sample scores, feeding type, and season were represented in plots in order to visualize the relation between sensory attributes and the sample distribution regarding these attributes. In order to make the plot visualization easier, each sample was represented by a unique point calculated by averaging the coordinates of the three replications.

Next, a PC‐ANOVA was run with IBM SPSS Statistics 24.0 to check the significance of the axes from PCA using the coordinates of each sample (observations, in rows) over the PCs (in columns), as described by Luciano and Næs (2009). An agglomerative hierarchical clustering was also done to confirm the distribution of the samples observed in the PCA according to season and feeding. Clustering analysis was based on the analysis of dissimilarities by the Euclidean distance and Ward's method, where two classes were predefined.

## 3 RESULTS

### 3.1 Attributes describing and differentiating lamb meat samples

**FIGURE 4:**
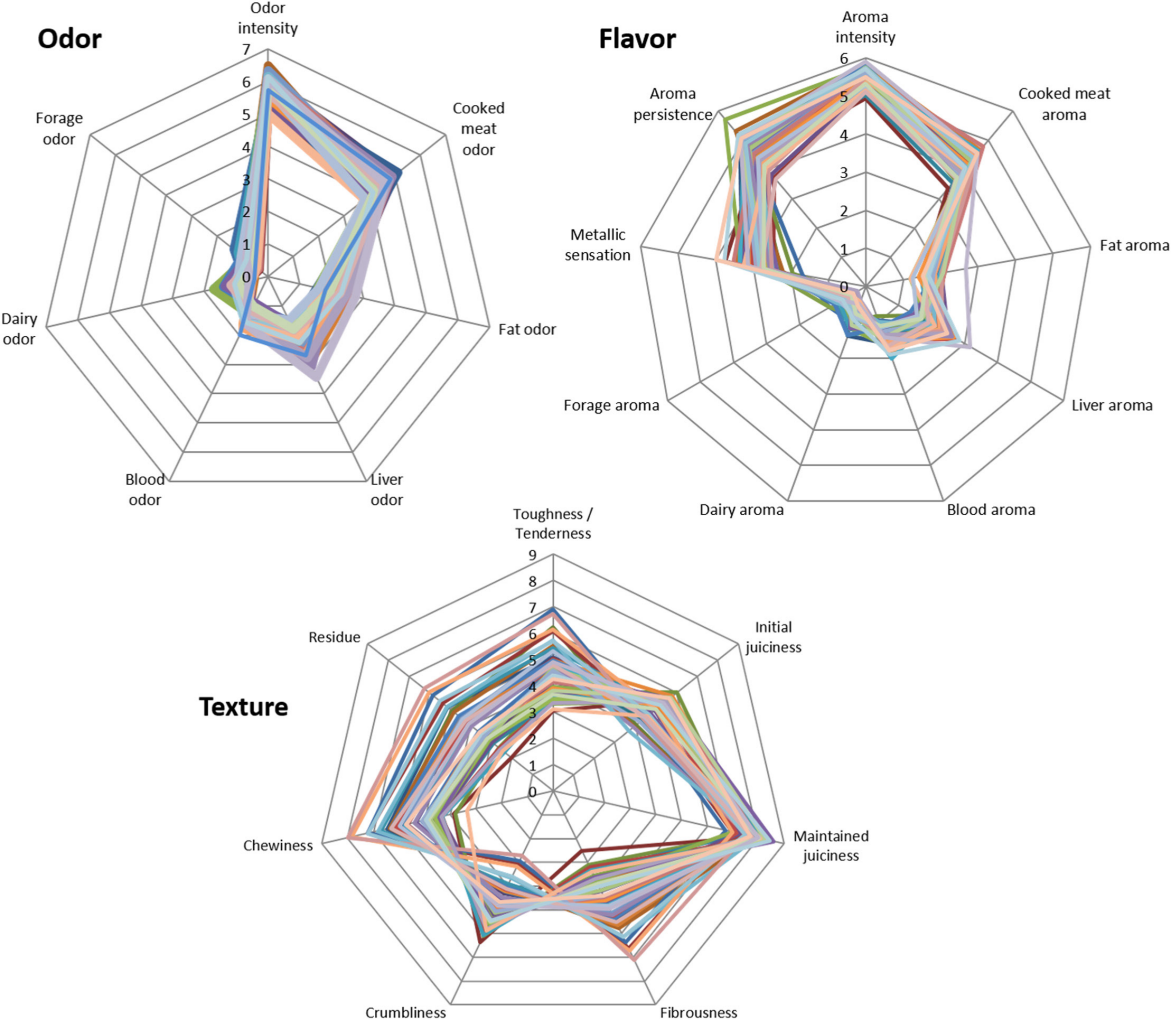
Panel mean scores of the 48 samples for odor, texture, and flavor attributes.

Panel mean scores for odor, texture, and flavor attributes of the 48 samples studied are shown in Figure 4. Regarding odor attributes, overall, odor intensity and cooked meat odor were the attributes with higher scores, values ranging from 5.01 to 6.48 and from 3.79 to 5.13, respectively. Liver and fat odor were the attributes with the next higher scores (from 1.50 to 3.43 and from 1.55 to 2.78, respectively). Blood, dairy, and forage odors presented low intensities in the samples, with maximum mean scores of 1.99 for blood, 1.72 for dairy, and 1.34 for forage odors.

The distribution of mean scores for flavor attributes was quite similar to that of odor attributes. Overall, aroma intensity, aroma persistence, and cooked meat aroma presented the highest values (ranging from 4.94 to 5.90, from 3.67 to 5.72, and from 3.37 to 4.80, respectively). The next sensation with a higher mean score was metallic sensation, ranging from 1.67 to 3.97. Liver aroma was the next attribute in intensity, ranging from 1.37 to 3.17. Fat and blood aromas presented lower mean scores, ranging from 1.19 to 2.67 and from 0.82 to 2.02, respectively. Dairy and forage aromas were the flavor attributes with the lowest mean scores (maximum mean scores of 1.37 and 1.10, respectively).

In the case of texture attributes, the range of the scale among samples was, in general, wider compared to odor and flavor attributes, as the mean scores for many of the attributes evaluated were high. How long juiciness was maintained (“maintained juiciness”) was the attribute with highest values (6.71 to 8.58), indicating that the juiciness of the samples after being chewed 10 times was generally very similar to its initial juiciness. The other texture attributes presented mean scores around the middle of the scale, and there was greater dispersion than maintained juiciness, with values ranging from 3.36 to 7.99 for chewiness, from 2.52 to 7.13 for fibrousness, from 3.05 to 6.91 for toughness, from 2.70 to 6.38 for crumbliness, from 2.04 to 6.24 for residue, and from 3.68 to 6.01 for initial juiciness.

Table 3 shows the F and *p* values from the ANOVA run to study the attributes' suitability for differentiating the various samples. Eighteen of the 23 attributes significantly differentiated (*p* ≤ .05) the samples. Blood odor and aroma, maintained juiciness, aroma intensity, and liver aroma were the only attributes without significant differences among the samples. Significant (*p* ≤ .05) interactions between assessor and sample were found for most of the odor and flavor attributes (except for blood odor and aroma, and metallic sensation), but not for texture attributes, indicating a considerably higher agreement among assessors when scoring texture attributes compared to odor and flavor attributes.

**TABLE 3:** *F* statistics and *p* values from ANOVA analysis of assessor scores for attributes evaluated in lamb meat samples.

Attribute	Sample(season)		Season		Session(season)		Assessor		Assessor*sample(season)	
	*F*	*p*	*F*	*p*	*F*	*p*	*F*	*p*	*F*	*p*
**Odor intensity**	1.422	.038	5.543	.035	3.987	0.000	23.352	0.000	1.370	0.000
**Cooked meat odor**	1.509	.019	2.566	.133	3.519	0.000	28.433	0.000	1.348	0.000
**Fat odor**	1.529	.016	7.367	.016	1.669	0.083	44.346	0.000	1.241	0.003
**Liver odor**	1.654	.005	13.478	.002	1.808	0.055	26.072	0.000	1.148	0.038
Blood odor	0.886	.687	5.035	.055	2.377	0.009	40.364	0.000	1.064	0.214
**Dairy odor**	2.117	.000	6.575	.020	3.525	.000	15.729	.000	1.398	.000
**Forage odor**	1.404	.044	19.146	.001	1.276	.239	24.731	.000	1.254	.002
**Toughness**	11.357	.000	0.189	.666	3.993	.000	4.107	.000	0.902	.903
**Initial juiciness**	2.142	.000	0.531	.480	5.668	.000	22.118	.000	0.943	.768
Maintained juiciness	1.202	.174	1.495	.249	1.602	.101	45.553	.000	1.100	.111
**Fibrousness**	7.751	.000	0.248	.621	3.832	.000	15.230	.000	0.984	.580
**Crumbliness**	8.074	.000	0.104	.749	3.450	.000	18.326	.000	0.990	.549
**Chewiness**	7.017	.000	0.010	.923	4.482	.000	28.868	.000	1.123	.069
**Residue**	9.012	.000	0.109	.743	3.315	.000	14.454	.000	1.011	.441
Aroma intensity	1.100	.306	2.861	.119	2.677	.003	47.665	.000	1.337	.000
**Cooked meat aroma**	1.658	.005	2.392	.145	3.727	.000	60.199	.000	1.278	.001
**Fat aroma**	1.908	.000	0.402	.535	3.486	.000	33.591	.000	1.277	.001
Liver aroma	1.299	.094	9.426	.010	2.760	.002	20.131	.000	1.168	.023
Blood aroma	1.193	.184	6.886	.025	1.812	.055	41.763	.000	1.135	.052
**Dairy aroma**	1.438	.033	14.521	.002	2.710	.003	13.825	.000	1.253	.002
**Forage aroma**	1.443	.032	14.480	.002	1.802	.056	29.737	.000	1.163	.026
**Metallic sensation**	1.602	.008	0.513	.488	5.030	.000	33.292	.000	1.047	.279
**Aroma persistence**	1.870	.001	9.918	.006	2.171	.018	31.883	.000	1.251	.002

*Note*: Attributes with significant differences (*p* ≤ .05) among samples are marked in bold.

### 3.2 Sensory differences regarding commercially declared feeding and sampling season

ANOVA was run with all data except data obtained from samples collected in stores where declared feeding changed from one season to the other (samples 10‐1NS and 10‐2S; 11‐1S and 11‐2NS; 18‐1NS and 18‐2S). Samples from store 15, although non‐suckling lamb in both seasons, were quite different from season 1 (approximately one year old) to season 2 (9–10 kg carcass weight; 2–3 months of age) (Table 1). They were removed from the analysis after checking that these samples changed from season 1 to season 2 in the opposite direction of the general trend. Results of ANOVA analyses of the remaining 40 samples are shown in Table 4.

**TABLE 4:** Mean scores, standard deviations (*SD*), *F* statistics, and *p* values from ANOVA analysis of panel scores for attributes evaluated in lamb meat samples, according to feeding and season factors.

Attribute	Feeding						Season						Feeding*season	
	Non‐suckling lamb		Suckling lamb		*F*	*p*	Season 1		Season 2		*F*	*p*	*F*	*p*
	Mean	*SD*	Mean	*SD*			Mean	*SD*	Mean	*SD*				
Odor intensity	5.88	0.483	5.89	0.367	0.459	.507	**6.00**	**0.402**	**5.77**	**0.445**	**5.066**	**.049**	1.777	.186
Cooked meat odor	4.45	0.481	4.36	0.477	0.179	.677	**4.23**	**0.426**	**4.60**	**0.460**	**11.526**	**.007**	**7.769**	**.007**
Fat Odor	2.08	0.469	2.04	0.450	0.117	.736	1.94	0.425	2.18	0.463	4.345	.065	0.510	.477
Liver odor	2.27	0.616	2.21	0.535	0.518	.481	**1.93**	**0.475**	**2.56**	**0.506**	**52.043**	**.000**	0.174	.677
Blood odor	1.37	0.385	1.34	0.434	0.281	.603	1.24	0.410	1.48	0.361	4.283	.066	0.029	.865
Dairy odor	0.83	0.411	0.90	0.475	1.728	.205	**1.05**	**0.450**	**0.66**	**0.327**	**14.722**	**.004**	0.068	.795
Forage odor	0.75	0.307	0.68	0.307	2.230	.153	**0.86**	**0.306**	**0.58**	**0.240**	**36.278**	**.000**	0.467	.496
Toughness	**4.96**	**1.029**	**4.02**	**0.843**	**12.278**	**.003**	4.50	1.079	4.66	1.048	0.483	.504	0.116	.734
Initial juiciness	**4.79**	**0.955**	**5.41**	**0.609**	**12.040**	**.003**	5.03	1.023	5.05	0.730	0.026	.876	0.805	.372
Maintained juiciness	7.67	0.541	7.77	0.594	1.853	.190	7.78	0.644	7.63	0.462	1.099	.320	0.180	.673
Fibrousness	**5.12**	**1.168**	**4.10**	**0.983**	**12.465**	**.002**	**4.41**	**1.157**	**5.01**	**1.185**	**6.708**	**.028**	0.319	.574
Crumbliness	**4.44**	**0.984**	**5.24**	**0.835**	**8.766**	**.008**	**4.98**	**0.968**	**4.54**	**1.002**	**5.364**	**.045**	0.251	.617
Chewiness	**5.84**	**1.301**	**4.93**	**0.935**	**8.523**	**.009**	5.30	1.169	5.66	1.308	3.015	.116	0.224	.637
Residue	**4.24**	**1.157**	**3.36**	**0.769**	**9.435**	**.007**	3.77	1.079	4.01	1.126	1.040	.334	1.098	.298
Aroma intensity	5.51	0.342	5.47	0.355	0.004	.950	5.56	0.357	5.42	0.324	3.023	.114	0.124	.726
Cooked meat aroma	4.23	0.465	4.09	0.528	0.830	.374	4.00	0.482	4.35	0.443	3.325	.099	**4.550**	**.036**
Fat aroma	1.77	0.464	1.77	0.399	0.013	.910	1.73	0.425	1.81	0.448	0.195	.668	1.995	.161
Liver aroma	2.14	0.517	2.09	0.602	0.071	.793	**1.87**	**0.511**	**2.37**	**0.471**	**19.338**	**.001**	2.403	.125
Blood aroma	1.40	0.449	1.42	0.461	0.028	.869	**1.28**	**0.455**	**1.54**	**0.412**	**7.426**	**.022**	0.074	.787
Dairy aroma	0.74	0.347	0.78	0.421	0.515	.482	**0.94**	**0.392**	**0.58**	**0.264**	**24.061**	**.001**	0.372	.543
Forage aroma	0.61	0.251	0.53	0.275	2.367	.141	**0.70**	**0.268**	**0.46**	**0.201**	**19.708**	**.001**	0.953	.332
Metallic sensation	2.88	0.650	2.91	0.669	0.042	.840	2.74	0.681	3.05	0.594	3.693	.084	**4.222**	**.043**
Aroma persistence	4.55	0.627	4.65	0.663	1.373	.257	**4.80**	**0.643**	**4.38**	**0.571**	**9.117**	**.014**	0.987	.323

*Note*: Mean, *SD*, *F*, and *p* values in bold indicate significant difference (*p* ≤ .05) for this attribute and factor.

Regarding declared feeding type, no differences in odor or flavor attributes were found between meat sold as suckling lamb and meat sold without this designation. In contrast, differences were found for all the texture attributes, except for maintained juiciness. Lambs sold as suckling lambs presented a significantly higher initial juiciness and crumbliness and significantly lower scores for toughness, fibrousness, chewiness, and residue. This suggests that meat sold as suckling lamb presents more desirable characteristics although limited to texture attributes.

Season had a significant effect on several attributes, mainly related to odor and aroma. Samples in season 1 had higher scores for odor intensity, dairy and forage odor and aroma, and for aroma persistence. In contrast, liver odor and aroma and blood aroma were significantly higher in season 2. Season also affected two texture attributes: fibrousness was higher in season 2, whereas crumbliness was higher in season 1.

Significant feeding*season interaction was found for three attributes: cooked meat odor and aroma, and metallic sensation. For these three attributes, the intensities increased from season 1 to season 2 for both types of feeding, but the increase was more marked for meats sold as suckling lamb. For cooked meat odor and metallic sensation, meat from suckling lamb had lower mean scores (4.07 and 2.65, respectively) than non‐suckling lamb meat (4.33 and 2.80, respectively) in season 1, which was the opposite in season 2 (4.65 and 3.18, respectively, in suckling lamb and 4.57 and 2.96, respectively, in non‐suckling lamb). This is mainly explained by the intensities increasing from season 1 to season 2 in all the suckling lamb samples, while three and four samples of non‐suckling lambs presented lower scores in season 2 for cooked meat odor and metallic sensation respectively. In the case of cooked meat aroma, the mean scores were higher in both seasons 1 and 2 for non‐suckling lambs compared to suckling lambs (4.09 vs. 3.86, respectively, in season 1, and 4.38 vs. 4.31, respectively, in season 2), but the increase in the intensity of this attribute from season 1 to season 2 was higher in the case of suckling lamb samples.

**FIGURE 5:**
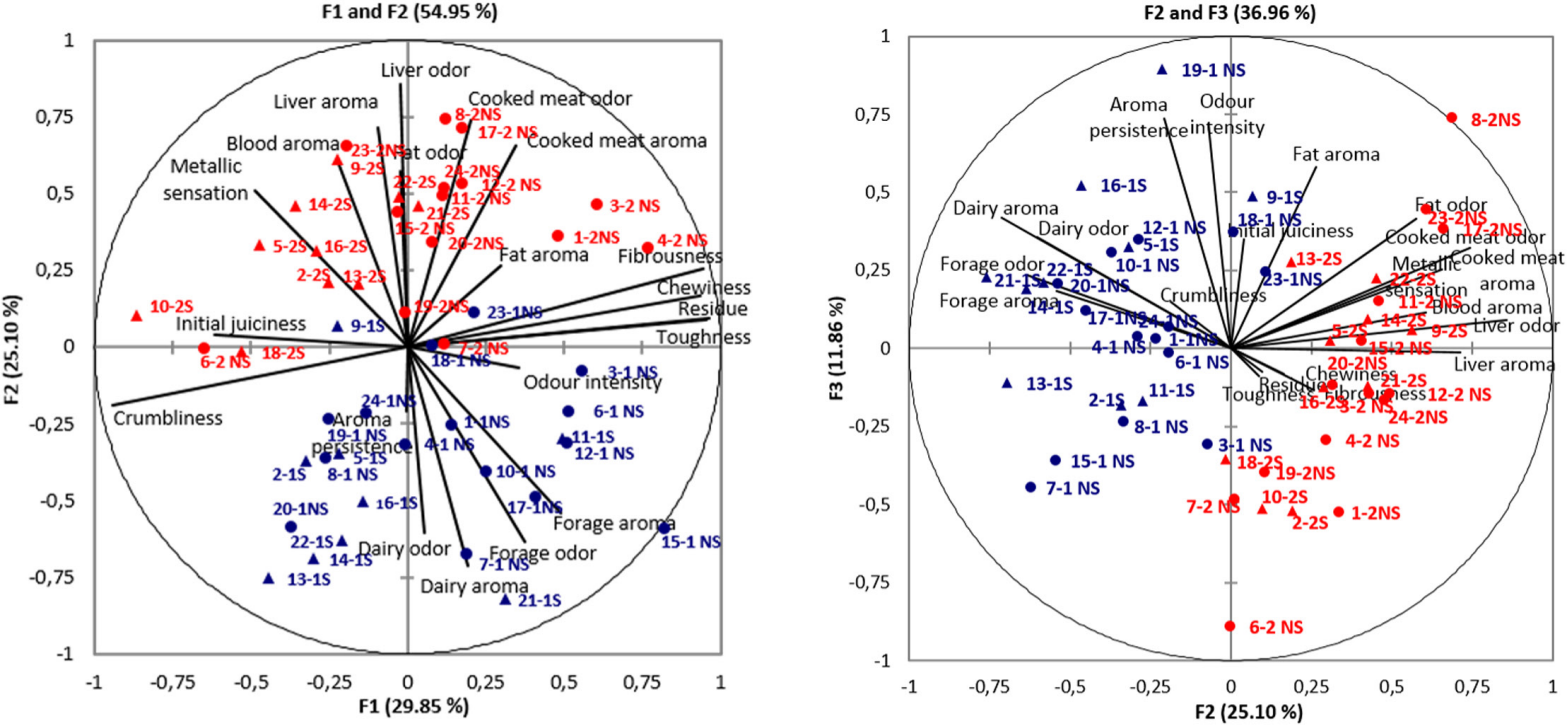
PCA plot of samples and sensory attributes. Triangles and circles represent suckling and non‐suckling lamb meat samples, respectively. Blue and red represent first and second season samples, respectively.

In order to be able to visualize the relation between attributes, a PCA was performed. Principal axes were drawn, and the projection of the samples was laid over them (Figure 5). This PCA did not include the three attributes (blood odor, maintained juiciness, and aroma intensity) that were not valid for distinguishing samples or groups according to season and feeding. Variance explained by the first two components was 54.95%. The third component accounted for an additional 11.86%. The first component accounts mainly for variance related to texture attributes, whereas the second and third components account for variance of odor and flavor attributes, with marginal contribution to explain variance of texture. Along the first component, crumbliness and initial juiciness appear as being contrary to fibrousness, chewiness, residue, and toughness. The latter four attributes also show greater correlation between them.

The second component separates samples according to odor and flavor attributes, ranging from dairy and forage character (odor and aroma) to liver odor and aroma, blood aroma, metallic sensation, and cooked meat odor and aroma.

With respect to sample characterization due to declared feeding, samples from suckling lambs (indicated as triangles) are mainly placed in the left part of Figure 4a, corresponding to samples with higher initial juiciness and crumbliness. Samples from non‐suckling lambs (indicated as circles) presented a higher dispersion in the plot, but with a clear trend to be placed more on the right side (tougher and more fibrous meat, with more residue and with more chewing required).

Regarding the effect of season, a clear separation can be observed in both Figure 4a and Figure 4b between samples from season 1 (in blue) and samples from season 2 (in red). Up‐down (Figure 4a) and left–right (Figure 4b) separation along the second component shows that sensory differences between seasons 1 and 2 are related to odor and flavor characteristics. Samples of season 1 (purchased in May) are characterized by dairy and forage aroma and, to some extent, by a higher odor intensity and longer aroma persistence. In contrast, samples of season 2 (December) are characterized mainly by liver characteristics, blood aroma, and cooked meat odor.

Coordinates of samples on the first three axes were submitted to ANOVA to check the significance of the axis from PCA. Results are shown in Table 5. The effect of feeding was significant for PC1, supporting the differences visualized between suckling and non‐suckling meat samples. The effect of season was significant for PC2 and PC3, and it confirmed the differences between samples from season 1 to season 2, which were related to odor and flavor attributes. No interactions for Season*Feeding were found for any of the PCs.

**TABLE 5:** *F* statistics and *p* values from ANOVA analysis of sample coordinates on PCs 1, 2, and 3 (PC‐ANOVA) according to feeding and season factors.

	Feeding		Season		Season*feeding	
	*F*	*p*	*F*	*p*	*F*	*p*
PC1	**10.888**	**.004**	0.340	.573	0.149	.700
PC2	0.000	.984	**34.291**	**.000**	3.873	.052
PC3	3.590	.074	**22.750**	**.001**	0.012	.912

*Note*: *F* and *p* values in bold indicate significant difference (*p* ≤ .05) for this PC.

The results from the agglomerative hierarchical clustering confirmed the sample distribution observed in the PCA plot (Figure 5), and the greater importance of texture attributes in comparison to odor and flavor attributes to classify the samples. In fact, 17 of the 19 samples from meat sold as suckling lamb were grouped in the same cluster (cluster 2, together with 16 samples from non‐suckling lambs), with only two samples sold as suckling lamb (11‐1S and 21‐1S) in cluster 1 (together with 13 samples from non‐suckling lambs). These two samples, 11‐1S and 21‐1S, are the most different from the other suckling lamb samples in the plot from PC1 and PC2 (the only samples placed in the fourth quadrant).

Regarding sample clustering according to season, samples from season 1 were classified relatively equally in cluster 1 (11) and cluster 2 (13). However, samples from season 2 were mostly classified in cluster 2 (20), with only four samples in cluster 1 (1‐2NS, 3‐2NS, 4‐2NS, and 7‐2NS; samples more explained by “hard” texture attributes than by odor/flavor attributes in PCA plot).

## 4 DISCUSSION

### 4.1 Attributes differentiating samples

Sensory attributes selected were suitable to describe lamb meat samples and to differentiate them and between groups according to season and declared feeding. However, maintained juiciness, although valid for describing samples, did not discriminate between samples or groups of samples, so it could be removed in future studies with these kinds of samples. The other texture attributes proved suitable for describing and discriminating between samples. In fact, texture attributes contributed to explaining the variability among samples more than odor and flavor attributes (Figure 3). Oltra et al. (2015), in a study on grilled lamb loins, reported a first component clearly determined by texture, which accounted for 81.5% of the total variation, while the second component only accounted for 7% of the variation. In a similar way, in a sensory study on different lamb meats, Gasperi et al. (2005) found that the dimension explained by three attributes of texture accounted for 45% of the variance, while the dimension related to 13 attributes of flavor accounted for 38% of the variance.

Within texture, the opposite location of initial juiciness and crumbliness, on the one hand, and fibrousness, chewiness, residue, and toughness, on the other hand, is very evident, as is the close relationship between these four last characteristics. This opposite relationship has been previously reported in other studies about lamb meat (Gasperi et al., 2005; Oltra et al., 2015; Ruiz de Huidobro et al., 2001).

Regarding odor and flavor, many of the selected 16 attributes were valid to discriminate between samples or groups of samples (Tables 3 and 4). Aroma intensity and blood odor were not discriminative, but odor intensity and blood aroma contributed to differentiating between samples and/or between sample groups. Some of the attributes used are discriminative between samples but not among sample groups (fat odor, cooked meat aroma, fat aroma, and metallic sensation) or vice versa (liver and blood aroma). The greater number of non‐discriminant aroma attributes compared to odor attributes (three non‐discriminant aroma attributes among samples vs. one non‐discriminant odor attribute; three non‐discriminant aroma attributes between sample groups vs. two non‐discriminant odor attributes) can be due, to a certain extent, to the greater difficulty in perceiving aroma attributes when, at the same time, other kinds of sensations are also perceived (texture and sapid and trigeminal sensations). In a study on sheep meat, Rousset‐Akrim et al. (1997) reported that assessors discriminated more clearly by smelling than tasting because there was a higher number of significantly discriminative attributes for odor than for flavor (7 versus 4, respectively), which suggested that smaller differences between samples were better distinguished by smelling.

### 4.2 Sensory differences related to declared feeding

Sensory characteristics of meat are determined by several ante‐ and post‐mortem factors, such as conservation and cooking (Sañudo et al., 2013; Xiong et al., 1999). The objective of the present survey‐based work is not to study the effect of these factors on meat sensory characteristics but to describe the samples marketed. In addition, sensory differences related to season and feeding type are described but without considering the specific feeding (of the non‐suckling lambs).

There are many studies about the effect of diet or feeding system on sensory characteristics of lamb meat, although few of them include the comparison between suckling and non‐suckling lamb meats. In any case, sensory attributes affected by the lambs' feeding vary considerably according to the study, and the effects on specific sensory attributes in various studies are often contradictory.

Meat sold under the “suckling lamb” denomination did not present any odor or flavor difference in comparison to lamb meat sold without this denomination. Panea et al. (2013) did not find a clear differentiation regarding sensory characteristics (lamb odor and flavor, fat flavor) between suckling and light lambs (22–24 kg) fed on grass and supplements. In fact, other factors such as breed or diet were more relevant to influence sensory characteristics. Gorraiz et al. (2000) described more woolly aromas and flavors and a more intense aftertaste for heavier lambs compared with suckling lambs. These differences in aroma and flavor could be due to the greater weight of the former, since the stronger flavor characteristics of meat from heavier lambs have been related to their higher intramuscular fat content (Horcada, 1996), which would be responsible for the development of several aromas when cooked (Brennand & Lindsay, 1992). On the other hand, other authors have described a greater intensity of several flavor attributes in suckling lambs. Carlucci et al. (1999) described meat from lambs exclusively fed with milk (slaughtered at 45 days) as higher in meaty odor and flavor than older lambs (slaughtered at 90 days) fed on commercial pellets. Gasperi et al. (2005), in a study on 12 different lamb meat samples where two of them were lamb fed exclusively on ewe's milk, found that these two samples were related to lamb odor, fat aroma, and milk aroma, and opposite to liver characteristics.

Regarding texture, all the texture attributes evaluated were significantly (*p* ≤ .05) different between the two sample groups, with the exception of maintained juiciness. Suckling lambs presented higher initial juiciness and crumbliness and lower toughness, fibrousness, chewiness, and residue than meat sold as non‐suckling lamb. Juiciness and tenderness have been traditionally described as the main texture drivers of sensory quality. According to this assumption, meat sold as suckling lamb presented better sensory characteristics in terms of texture.

Onega (2003) found suckling lamb meat to be juicier than meat from heavier/older lambs (“Ternasco” from Aragón). The increased initial juiciness of meat sold as suckling lamb could be explained by the greater amount of water released by these samples during the initial chewings, as described by Hawkins et al. (1985). This difference could be associated with the animal's age and weight, since meat sold without the “suckling lamb” denomination came from older and, therefore, heavier lambs. Indeed, several authors have related the decrease in juiciness to an increase in lamb weight (Gorraiz et al., 2000; Solomon et al., 1980). On the other hand, the carcass fatness, which increases with age, has been positively correlated with juiciness *(*Mushi et al., 2008; Priolo et al., 2002 *)* and tenderness (Fisher et al., 2000; Mushi et al., 2008; Priolo et al., 2002). Considering these opposed effects, it could be hypothesized that, in the present study, the capacity to release water when chewing would be of greater importance than the contribution of intramuscular fat to the sensation of “juiciness.”

The lower values of meat sold as suckling lamb for toughness, fibrousness, chewiness, and residue could be related to the lightweight of lamb carcasses. Onega (2003) found suckling lambs to be tenderer than heavier lambs (“Ternasco” from Aragón). Miguel et al. (2021) found meat from heavy carcasses (20 and 25 kg) tougher and springier than meat from light carcasses (10 and 15 kg) in a study on lambs fed with milk and concentrate. Gorraiz et al. (2000) found meat from light lambs (60 days old and 24 kg live weight) tougher and more difficult to swallow than meat from suckling lambs (25 days old and 12 kg live weight). This negative relationship between age and weight of lambs and meat tenderness has been previously described by other authors too (Brewer et al., 1987; Ouali, 1991). However, Carlucci et al. (1999) found higher values of cohesiveness and stringiness (fibers perceived during chewing) and lower scores for tenderness and juiciness in suckling lambs in comparison with older lambs, suggesting the higher fat content of older animals as the possible explanation. Panea et al. (2013) did not find differences in tenderness and juiciness between meat from suckling lambs (12 kg) and meat from light lambs (24 kg) fed on grass and supplements.

The higher fibrousness, chewiness, and residue and the lower crumbliness of non‐suckling lamb samples are directly related to the toughness of these samples, and it could be explained by differences in several physicochemical parameters such as insoluble collagen content, fat content, muscle fiber length, or degree of dehydration during cooking (Čandek‐Potokar et al., 1998). Compared to juiciness and toughness, other texture attributes are scarcely mentioned in suckling versus non‐suckling lamb studies, and no clear references about their contribution to differentiate between samples were found.

As for odor and flavor attributes, it seems that lamb weight would be a more decisive factor to modulate texture attributes than feeding lambs exclusively with milk or not.

### 4.3 Sensory differences related to season

Lamb meat samples bought at different moments of the year showed several significant differences (*p* ≤ .05). Samples purchased in May had higher scores for odor intensity, dairy and forage odor and aroma, aroma persistence, and crumbliness, while samples collected in December received higher scores for liver odor and aroma, blood aroma, and fibrousness. In this sense, although sensory quality aspects are not addressed in this study, sensory characteristics seem to be more appropriate in lamb meat samples surveyed in May (season 1) in comparison to lamb meat samples surveyed in December (season 2), which presented a more marked liver and blood character (not very desirable) in addition to being more fibrous and less crumbly.

There are few studies comparing lamb meat sensory characteristics across different seasons, and no studies which focused on young lambs have been found.

The factor of season is predominantly related to animal feeding. However, as samples came from a wide survey, there is no exhaustive information about this factor. In terms of the effect of season on sensory characteristics, in a study on meat from lambs of the Rasa aragonesa breed slaughtered approximately 100 days into winter (January) and in summer (June–July), Miranda de la Lama et al. (2012) found that meat from lambs slaughtered in winter presented higher lamb odor and fat flavor, but lower juiciness, liver flavor, and metallic flavor than meat from lambs slaughtered in summer. None of these differences were found in the present study and, in the case of liver flavor, the result was the opposite.

Regarding the effect of feeding on lamb meat sensory characteristics, the flavor profile could be largely related to diet and the intramuscular fat content and profile (Ådnøy et al., 2005; Duckett & Kuber, 2001; McCaughey & Clipef, 1995; Resconi et al., 2009). In addition, the ewe's diet determines the milk composition and, indirectly, the fatty acid profile of the meat of suckling lambs (Battacone et al., 2021; Revilla et al., 2021). However, results of different studies regarding lamb feeding are contradictory. Several authors have not found any difference between animals fed with grass compared to concentrate (Panea et al., 2013; Pethick et al., 2005; Solomon et al., 1996), whereas other authors have observed more intense odor and flavor in meat from animals fed on grass in comparison to animals fed on concentrate (Angood et al., 2008; Rousset‐Akrim et al., 1997). The higher content of polyunsaturated fatty acids observed in meat from animals fed with grass could explain these greater odor and flavor intensities (Fisher et al., 2000; Gkarane et al., 2019). A similar trend was reported in beef cattle meat by Priolo et al. (2001), as greater forage and milky characteristics were found in meat from animals fed on grass. In this sense, it can be hypothesized that a probable greater pasture proportion in the diet of non‐suckling lambs in May (season 1) in comparison to December (season 2) could explain the greater intensities observed in our study for global odor as well as for forage odor and aroma.

Grass feeding has also been related to liver odor and flavor (Priolo et al., 2002). However, in the present study, samples collected during season 2 (in which non‐suckling animals were likely fed, at least partially, with concentrates) presented higher liver odor and aroma than meat collected in season 1.

The reason for texture differences observed in the present study (season 1 higher in crumbliness and lower in fibrousness compared to season 2) is not so apparent. In fact, the influence of diet on texture would be insignificant or non‐existent (Field et al., 1978; Hopkins & Nicholson, 1999; Kemp et al., 1981; Summers et al., 1978; Vipond et al., 1995).

## 5 CONCLUSIONS

Overall, the selected sensory attributes proved appropriate in describing the samples of the study as well as in discriminating between them. The “maintained juiciness” attribute could be removed in future studies in order to simplify the evaluation process, since it did not discriminate between the various samples. This study also provides sensory references for each attribute that can be useful for future studies on lamb meat.

Regarding sample description, the odor and flavor attributes with greater intensities were odor and aroma intensity, aroma persistence, and cooked meat odor and aroma; while fat, liver, and blood odor and aroma, and metallic sensation, presented lower intensities. Forage and dairy odor and aroma were recorded as having the lowest intensities. In any case, all of these attributes were suitable for differentiating between individual samples and/or between groups (suckling vs. non‐suckling lamb, season 1 vs. season 2).

Lamb meat sold as suckling lamb in the different cities of the Basque Country and Navarre did not differ from other lamb meat sold in the same stores in terms of odor and flavor characteristics. On the contrary, there were clear differences between these two types of meats in terms of texture, where meat sold as suckling lamb was tenderer, juicier, with higher crumbliness, and with lower fibrousness, chewiness, and residue compared to meat sold as non‐suckling lamb. From these results, it can be interpreted that samples of the study sold as suckling lamb can be classified as having more appreciated texture characteristics.

Several sensory characteristics, mainly related to odor and flavor, varied according to the season of the year, which could be related to variations in the diet of non‐suckling lambs throughout the year (likely from pasture‐based in spring to concentrate‐based in autumn).

This study was based on a survey performed to characterize the lamb meat sold in Basque and Navarre regions, while lamb denomination (suckling or non‐suckling) and season effects have been considered. However, it was not possible to collect any other data that could help in a more exhaustive explanation of the results obtained. This could be considered as a limitation per se, although a controlled trial would have noticeably diminished the sample number, while not being as representative of the meat commercialized in the surveyed regions as this study was.

## AUTHOR CONTRIBUTIONS

**Iñaki Etaio:** Conceptualization (lead); data curation (lead); formal analysis (equal); investigation (equal); methodology (equal); writing – original draft (lead); writing – review and editing (lead). **Leire Bravo‐Lamas:** Formal analysis (equal); investigation (supporting); methodology (equal); writing – original draft (supporting). **Francisco José Pérez‐Elortondo:** Conceptualization (equal); data curation (supporting); formal analysis (equal); funding acquisition (equal); investigation (equal); methodology (equal); project administration (equal); resources (equal); supervision (equal); writing – original draft (supporting). **Luis Javier R. Barron:** Conceptualization (supporting); data curation (equal); funding acquisition (lead); investigation (equal); project administration (lead); resources (equal); writing – original draft (supporting). **Noelia Aldai:** Conceptualization (equal); formal analysis (equal); funding acquisition (supporting); investigation (equal); methodology (equal); project administration (equal); supervision (lead); writing – original draft (equal); writing – review and editing (equal).

## CONFLICT OF INTEREST STATEMENT

The authors declare no conflict of interest.

## Data Availability

Data available on request from the authors.
